# Acupoint catgut embedding therapy for functional constipation: A protocol for systematic review and meta-analysis

**DOI:** 10.1097/MD.0000000000024286

**Published:** 2021-01-29

**Authors:** Fumin Wang, Man Jin, Yuanzhang Hu, Yuxuan Chao, Xiaoen Cheng, Yuan Gao

**Affiliations:** a School of Acupuncture-Moxibustion and Tuina; b School of Nursing; c School of Medical Information Engineering; d School of Clinical Medicine, Chengdu University of Traditional Chinese Medicine, Chengdu, China, Chengdu University of Traditional Chinese Medicine. No. 37 Shierqiao Road, Jinniu District, Chengdu, Sichuan, China.

**Keywords:** acupoint catgut embedding therapy, functional constipation, meta-analysis, protocol

## Abstract

**Background:**

This review will assess current evidence related to the effectiveness and safety of acupoint catgut embedding therapy for functional constipation (FC) and provide efficacy assessments for clinical applications.

**Methods:**

We will search the following databases for relevant trials: PubMed, EMBASE OVID, Cumulative Index of Nursing and Allied Health Literature, OVID MEDLINE, Web of Science, the Cochrane Central Register of Controlled Trials, Cochrane library, and Scopus. We will also search the following Chinese databases for trials published in the Chinese literature: China National Knowledge Infrastructure Database (CNKI), Chinese Scientific Journals Database, Wan Fang Database, Chinese Biomedicine and other resources from inception to December 2020. Only randomized controlled trials comparing acupoint catgut embedding versus acupuncture or sham acupuncture or placebo or other therapies will be included. The outcomes involved mean spontaneous bowel movements, complete spontaneous bowel movements, the Bristol Stool Form Scale, the Cleveland Clinic Score, Patient Assessment of Constipation symptom and so on. The risk of bias assessment and quality of evidence for outcomes will be appraised using the Cochrane Risk of Bias Tool and the Grading of Recommendations, Assessment, Development and Evaluation guidelines. RevMan 5.3 software will be employed for the meta-analysis.

**Results:**

This work will compare and arrange the comparative efficacy of acupoint catgut embedding with different treatments for FC by summarizing the current evidences.

**Conclusion:**

The results of this meta-analysis may help doctors determine the best treatments for patients to manage FC.

**Ethics and dissemination:**

This is a protocol with no patient recruitment and personal information collection, approval by the ethics committee is not required.

**OSF Registration number:**

DOI 10.17605/OSF.IO/XTKE2.

## 1 Introduction

Functional constipation (FC) is a common disease in clinical practice. It is characterized by infrequent bowel movements and other associated symptoms of difficult defecation, hard and lumpy stools, feeling of tension, a sensation of incomplete evacuation.^[1,2]^ The pathophysiology of functional constipation is multifactorial and associate with non-systemic diseases and dysmotility of the GI tract.^[3–5]^ Long-term chronic FC not only reduces the quality of life of the patients, but also causes problems of the genitourinary system, respiratory system, circulatory system, nervous system and other systems.^[6–10]^ It also causes serious harm to the mental and psychological health of the patients.^[11]^ FC affects over 14% of adults globally and has become one public health issue.^[12]^

There are 3 therapies for FC currently: nonpharmaceutical, pharmaceutical, and surgical.^[13]^ Biofeedback therapy, supplementing the diet with fiber, and exercising are only recommended for mild symptoms of constipation.^[14–17]^ Surgical treatment is not widely used in clinic practice because of its disadvantages such as large injury and uncertain clinical efficacy.^[18]^ At present, the clinical treatment of FC mostly adopts laxatives and gastrointestinal prokinetic drugs.^[19]^ In the short term, the frequency of spontaneous bowel movements can be increased, but the long-term efficacy is not good, and there are some toxic and side effects.^[20]^ Therefore, effective complementary and alternative therapies with few side effects for FC are important for patients.

Acupoint catgut embedding therapy is a therapeutic method established and developed on the basis of traditional acupuncture. Traditional acupuncture methods can only retain filiform needles for a short time, but acupoint catgut embedding has a long-term and lasting stimulation on acupoints for about 30 days. And catgut embedding needle is thicker than filiform needle, so it can produce stronger needle sensation. Some studies have found that continuous stimulation of catgut to related acupoints can enhance the contractility and excitability of intestinal smooth muscle and promote intestinal peristalsis. The clinical application of this approach achieved stable curative effects and satisfactory results in the treatment of chronic FC.^[21–24]^

Nowadays, acupoint catgut embedding is widely applied in the clinical practice of functional constipation, but there is no systematic review and meta-analysis that provides persuasive evidence of its effect on FC. This study will summarize the current evidences and conduct a systematic review and meta-analysis to assess the efficacy and safety of acupoint catgut embedding for patients with FC.

## 2 Methods

### 2.1 Registration of the review

Our protocol for this systematic review has been registered on the open science framework (OSF), the registration number is DOI 10.17605/OSF.IO/XTKE2 (https://osf.io/xtke2), and the protocol is designed strictly follow the Preferred Reporting Items for Systematic Reviews and Meta-Analyses protocols (PRISMA-P) statement guidelines.

### 2.2 Inclusion and exclusion criteria

#### 2.2.1 Types of studies

Randomized controlled trials comparing acupoint catgut embedding for FC with no treatment, placebo, or conventional drugs (eg, laxative agents) will be included. All eligible trials will be included regardless of language and publication types. Articles of the following research types will be excluded: case series, qualitative studies, case-control studies, observational studies, animal experiments, experiences, review articles. There are no restrictions on study area, race, patient age, and gender.

#### 2.2.2 Types of participants

Participants with FC over the age of 18 years will be considered in this study. The diagnosis of FC needs to be consistent with Rome III or IV criteria rather than other clinical research guidelines. Studies with participants that include pregnant women, lactating women, addicts, strokes will be excluded.

#### 2.2.3 Types of interventions

In the intervention group, patients received acupoint catgut embedding without restrictions of points and treatment courses. The trials that the intervention group is acupoint catgut embedding combines with other therapies should not be included. In the control group, patients received conventional drugs (eg, laxative agents), no treatment, sham or placebo acupoint catgut embedding, and etc. Sham acupoint catgut embedding or placebo will be classified into a category as inert control.

#### 2.2.4 Types of outcome measures

The primary outcomes will be the improvement in mean spontaneous bowel movements,complete spontaneous bowel movements at the end of all sessions and the Bristol Stool Form Scale, the Cleveland Clinic Score, Patient Assessment of Constipation symptom.

The secondary outcomes involved Patient Assessment of Constipation Quality of Life Questionnaire, Self-rating Anxiety Scale, Self-rating Depression Scale and Transit time measurement (radiopaque markers), functional rectoanal evaluation (proctoscopy, anorectal manometry, defecography, cinedefecography), or electromyography. And the number and types of adverse events will be assessed safety evaluation.

### 2.3 Data sources and search strategy

We will systematically search electronic database from inception to December 2020, including PubMed, EMBASE OVID,CINAHL,OVID MEDLINE, Web of Science, the Cochrane Central Register of Controlled Trials, Cochrane library, Scopus, CNKI,VIP, Wan Fang Database, CBM and other resources from inception to December 2020. The search terms will include intervention methods, diseases and research types: (‘acupoint catgut embedding’ or ‘acupoint catgut embedding therapy’ or ‘acupoint therapy’ or ‘thread-embedding acupoint ligation’ or ‘acupoint ligat thread embed’) and (‘functional constipation’ or ‘FC’ or ‘outlet obstruction constipation’ or ‘OOC’ or ‘slow transit constipation’ or ‘STC’ or ‘mixed functional constipation’) and (‘clinical trial’ or ‘randomized controlled trial’ or ‘randomized’ or ‘trial’). To avoid missing ongoing clinical trials, we will search the following two trial registration centers to identify relevant studies: China Clinical Trial Registry (www.chictr.org.cn/index.aspx), The US National Institutes of Health Ongoing Trials Register (www.clinicaltrials.gov), The World Health Organization International Clinical Trials Registry platform (www.who.int/trialsearch). Besides, we will screen the references of included studies to identify other potential clinical trials. The search strategy for PubMed is shown in Table 1. This search strategy will be slightly modified and used in several other databases.

**Table 1:** The search strategy in PubMed.

#1 Functional Constipation[Title/Abstract]
#2 “Constipation”[Mesh]
#3 Chronic constipation[Title/Abstract]
#4 Idiopathic constipation[Title/Abstract]
#5 Slow transit constipation[Title/Abstract]
#6 Functional defecatory disorder[Title/Abstract]
#7 Outlet obstruction constipation[Title/Abstract]
#8 Mixed functional constipation[Title/Abstract]
#9 OOC[Title/Abstract]
#10 STC[Title/Abstract]
#11 FC [Title/Abstract]
#12 #1 or #2or #3or #4or #5or #6or #7or #8or #9or #10or #11
#13 “Acupuncture”[Mesh]
#14 “Acupuncture Therapy”[Mesh]
#15 “Acupuncture Points”[Mesh]
#16 acupoint catgut embedding[Title/Abstract]
#17 acupoint catgut embedding therapy[Title/Abstract]
#18 acupoint therapy[Title/Abstract]
#19 thread-embedding acupoint ligation[Title/Abstract]
#20 acupoint ligat thread embed[Title/Abstract]
#21 #13 or #14or #15or #16or #17or #18or #19or #20
#22 Randomized controlled trial[Publication Type]
#23 Controlled clinical trial[Title/Abstract]
#24 Randomized[Title/Abstract]
#25 Randomly[Title/Abstract]
#26 #22 or #23or #24or #25
#27 #12 and #21 and #26

FC = functional constipation, OOC = outlet obstruction constipation, STC = slow transit constipation.

### 2.4 Data collection and management

#### 2.4.1 Data selection

**Figure 1:**
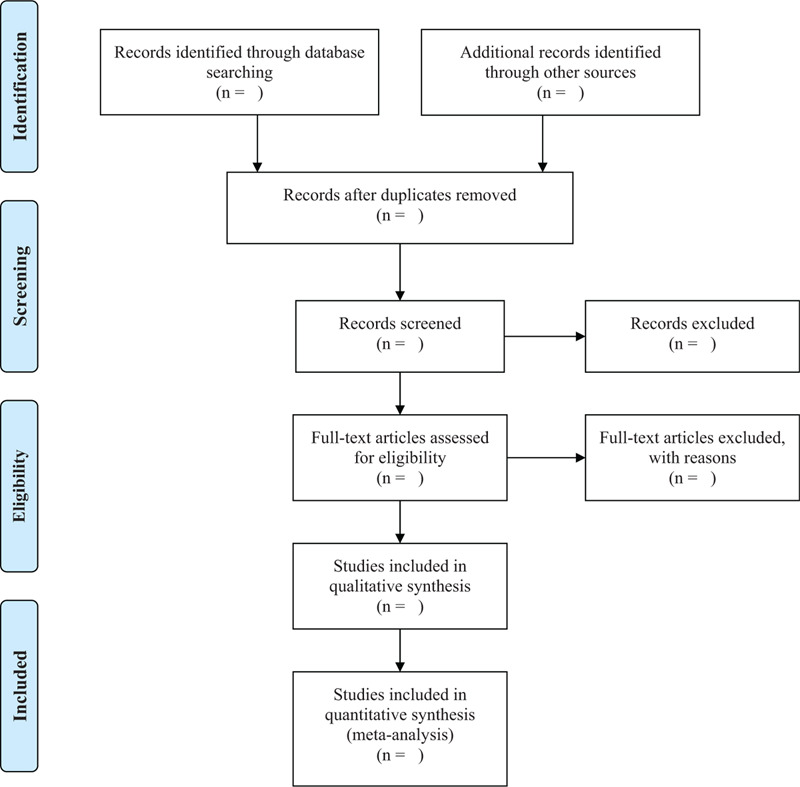
Flow diagram of the selection process.

First, use the EndNote X9 software to exclude duplicate references from different databases. Then, two reviewers (Fumin Wang and Man Jin) will evaluate the titles and abstracts of each clinical trial to identify the eligible studies independently. Finally, read the full text to assess eligible studies. If there is a disagreement, it will be resolved through discussions with the third reviewer (Xiaoen Cheng). Figure 1 shows the flow of this systematic review and meta-analysis.

#### 2.4.2. Data extraction

2.4.2

Two reviewers (Fumin Wang and Man Jin) will independently examine eligibility for inclusion in the study and use Microsoft Excel to encode and extract data based on pre-designed data extraction forms after reaching a consensus. The data will include the first author, country, year of publication, characteristics of participants, randomization, allocation concealment and blinding methods, interventions, acupoint selection, outcome measures, main outcomes, secondary outcomes, study duration, adverse effects, main conclusions, funding source, and conflicts of interest. Particularly, the third reviewer (Xiaoen Cheng) will take part in the discussion and decide to select the controversial search or not.

#### 2.4.3 Management of missing data

We will contact the researchers for the missing data by Email. If the missing data cannot be obtained, it will be excluded.

### 2.5 Assessment of risk of bias and reporting of study quality

Two review authors (Fumin Wang and Man Jin) will independently evaluate each included study and will follow the domain-based evaluation as developed by the Cochrane Handbook for Systematic Reviews of Interventions. They will assess the following domains: selection bias (random sequence generation and allocation concealment); performance bias (blinding of participants and personnel); detection bias (blinding of outcome assessment); attrition bias (incomplete outcome data); reporting bias (selective reporting); other bias (such as pre-sample size estimation, early stop of trial). Each domain will be divided into three categories: low risk, high risk, or unclear risk.

### 2.6 Measures of treatment effect

We will analyze the data with RevMan software (Version 5.3). Mean difference (MD) or standardized mean difference (SMD) with 95% confidence interval (95% CI) will be used to analyze continuous data. Risk ratio with 95% CI will be used to analyze dichotomous data.

### 2.7 Unit of analysis issues

The analysis will focus on patients in randomized controlled trials. If more than 1 objective is used, we will conduct separate multiple meta-analyses for each treatment arm. If multiple non-acupoint catgut embedding control groups are included, pooled analyses of the control groups against the intervention group will be used.

### 2.8 Assessment of heterogeneity

We will test the heterogeneity of data by calculating the value of *I*^2^ statistics and *x*^2^ test. *I*^2^ values of 25% is considered low levels of heterogeneity, 50% indicated moderate levels, and 75% indicated high levels. Since low or moderate heterogeneity (*P* > .1, *I*^2^ < 50%) suggests little variability among these studies, the data will be analyzed in a fixed-effects model. When significant heterogeneity occurs among the studies (*P* < .1, *I*^2^ > 50%), a random-effect model will be performed to analyze the data. At this very moment, the subgroup stratification analysis will be further carried out to explore the possible sources of heterogeneity.^[25,26]^

### 2.9 Assessment of reporting biases

Funnel plots is used to assess reporting biases. If funnel plot asymmetry is detected, the reasons will be analyzed.

### 2.10 Data synthesis

For continuous data, we will use mean difference or standard mean difference SMD to measure the therapeutic effect of 95% CIs. If significant heterogeneity is found, we will use the random- effects model instead. For dichotomous data, we will denote the outcomes as relative risks with 95% CIs. If the *I*^2^ test is < 50%, the fixed-effects model will be used for data synthesis. If the *I*^2^ test is between 50% and 75%, the random-effects model will be conducted for data synthesis. If the *I*^2^ test is higher than 75%, we will investigate possible reasons from both clinical and methodological perspectives, and provide a descriptive analysis or conduct subgroup analysis.

### 2.11 Subgroup or sensitivity analysis

Subgroup analysis will be conducted to evaluate the specific influence of intervention type, study quality, study location, treatment duration on pooled results. If the data is insufficient, qualitative synthesis will be conducted instead of quantitative synthesis. In addition, sensitivity analysis will be performed to examine the robustness of the results by eliminating low quality trials.

### 2.12 Sensitivity analysis

A sensitivity analysis will be performed according to the following criteria: sample size, heterogeneity qualities, and statistical model (random-effects or fixed-effects model).

### 2.13 Grading the quality of evidence

We will use the Grading of Recommendations Assessment, Development and Evaluation (GRADE) guidelines to assess the overall quality of evidence. The overall quality of the evidence for each outcome will be determined after considering each of these factors and graded as: high, moderate, low, very low.

## 3 Discussion

FC is a common health problem causing significant physical and emotional distress to patients and affects their quality of life. Acupoint catgut embedding therapy has been used in many diseases including FC as an adjuvant therapy at present. Our study will assess current evidence related to the effectiveness and safety of acupoint catgut embedding therapy in FC.

## Author contributions

**Conceptualization**: Fumin Wang.

**Data curation**: Man Jin, Yuanzhang Hu.

**Formal analysis**: Yuxuan Chao, Man Jin.

**Investigation**: Yuan Gao.

**Methodology**: Fumin Wang.

**Project administration**: Fumin Wang, Yuanzhang Hu.

**Supervision**: Xiaoen Cheng.

**Writing – original draft**: Fmin Wang, Man Jin.

**Writing – review & editing**: Yuan Gao, Xiaoen Cheng.

## Abbreviations

Abbreviation: FC = functional constipation.
